# Supplementary material for: Genetic Polymorphisms, Mediterranean Diet and Microbiota-Associated Urolithin Metabotypes can Predict Obesity in Childhood-Adolescence
Source: Sci Rep. 2020 May 12;10:7850. doi: 10.1038/s41598-020-64833-4 (PMC7217888; doi:10.1038/s41598-020-64833-4)
Supplement: Supplementary file 1 — Supplementary information. [file 41598_2020_64833_MOESM1_ESM.pdf]

Genetic Polymorphisms, Mediterranean Diet and Microbiota-Associated Urolithin Metabotypes can Predict Obesity in Childhood-Adolescence

Adrián Cortés-Martín, Gonzalo Colmenarejo, María Victoria Selma, and Juan Carlos Espín*

SNP	Gene		Related-gene processes	Related-gene disease	Genotype frequencies (%)[a]			*p*[b]
rs4343	*ACE*	Angiotensin I converting enzyme	Signalling, metabolism	CVD, MetS, Alzheimer	A/A 17.8	A/G 47.2	G/G 35.0	1.000
rs2241766	*ADIPOQ*	Adiponectin	Metabolism	MetS, diabetes, cancer	G/G 1.8	G/T 30.9	T/T 67.3	1.000
rs1501299	*ADIPOQ*	Adiponectin	Metabolism	MetS, diabetes, cancer	T/T 9.0	G/T 33.8	G/G 57.2	1.000
rs1801253	*ADRB1*	Adrenoceptor β1	Lipolysis and fat oxidation	CVD	G/G 16.2	C/G 25.9	C/C 57.8	0.000
rs4994	*ADRB3*	Adrenoceptor β3	Signalling, metabolism	MetS, diabetes	A/A 88.8	A/G 10.7	G/G 0.5	1.000
rs699	*AGT*	Angiotensinogen	Blood pressure	CVD	A/A 33.3	A/G 42.3	G/G 24.4	0.215
rs7913948	*ALOX5*	Arachidonate 5-lipoxygenase	Inflammation	CVD	A/A 2.3	A/G 29.4	G/G 68.3	1.000
rs5082	*APOA2*	Apolipoprotein A2	Metabolism	Obesity, CVD, diabetes	A/A 0.0	A/G 88.1	G/G 11.9	0.000
rs3813627	*APOA2*	Apolipoprotein A2	Metabolism	Obesity, CVD, diabetes	G/G 44.4	G/T 42.6	T/T 13.0	1.000
rs662799	*APOA5*	Apolipoprotein A5	Metabolism-triglycerides	CVD	A/A 84.9	A/G 14.8	G/G 0.3	1.000
rs693	*APOB*	Apolipoprotein B	Metabolism	MetS, diabetes, cancer	G/G 35.1	A/G 45.5	A/A 19.4	1.000
rs512535	*APOB*	Apolipoprotein B	Metabolism	Obesity, CVD, diabetes	C/C 29.9	C/T 47.1	T/T 23.0	1.000
rs7412	*APOE*	Apolipoprotein E	Metabolism	Alzheimer, CVD	T/T 1.0	C/T 11.7	C/C 87.3	1.000
rs429358	*APOE*	Apolipoprotein E	Metabolism	Alzheimer, CVD	T/T 82.8	C/T 16.2	C/C 1.0	1.000
rs708272	*CETP*	Cholesteryl ester transfer protein, plasma	Metabolism	CVD	A/A 14.6	A/G 44.7	G/G 40.7	1.000
rs3749474	*CLOCK*	Clock circadian regulator	Signalling, metabolism	Obesity, CVD	C/C 43.8	C/T 44.0	T/T 12.2	1.000
rs4580704	*CLOCK*	Clock circadian regulator	Signalling, metabolism	Obesity, MetS	C/C 39.7	C/G 50.5	G/G 9.8	0.922
rs1130864	*CRP*	C-Reactive protein	Inflammation	CVD	G/G 46.5	A/G 41.9	A/A 11.6	1.000
rs1799883	*FABP2*	Fatty acid-binding protein 2	Metabolism	Diabetes, MetS	C/C 51.9	C/T 37.3	T/T 10.8	1.000
rs9939609	*FTO*	Fat mass and obesity-associated	Metabolism	Obesity, CVD, MetS, diabetes	T/T 36.9	A/T 43.3	A/A 19.8	1.000
rs9935401	*FTO*	Fat mass and obesity-associated	Metabolism	Obesity, CVD, MetS, diabetes	G/G 36.4	A/G 42.9	A/A 20.7	1.000
rs9928094	*FTO*	Fat mass and obesity associated	Metabolism	Obesity, CVD, MetS, diabetes	A/A 31.7	A/G 43.4	G/G 24.9	1.000
rs9930333	*FTO*	Fat mass and obesity associated	Metabolism	Obesity, CVD, MetS, diabetes	T/T 32.3	G/T 44.2	G/G 23.5	1.000
rs8061518	*FTO*	Fat mass and obesity-associated	Metabolism	Obesity, CVD, MetS, diabetes	G/G 12.0	A/G 40.9	A/A 47.1	1.000
rs1260326	*GCKR*	Glucokinase regulatory protein	Metabolism of carbohydrates	CVD, diabetes	C/C 34.3	C/T 44.5	T/T 21.2	1.000
rs696217	*GHRL*	Ghrelin/obestatin prepropeptide	Signalling, metabolism	Obesity, cancer	T/T 1.0	G/T 13.6	G/G 85.4	1.000
rs5443	*GNB3*	(G protein), β-polypeptide 3	Signalling, metabolism	Obesity, CVD, diabetes	C/C 38.8	C/T 47.4	T/T 13.8	1.000
rs11554159	*IFI30*	Interferon γ-inducible protein 30	Inflammation	Cancer	A/A 9.8	A/G 35.3	G/G 54.9	0.173
rs1800896	*IL10*	Interleukin 10	Inflammation	CVD, obesity, cancer	C/C 13.6	C/T 42.6	T/T 43.8	1.000
rs1143634	*IL1B*	Interleukin 1β	Inflammation	Diabetes, periodontitis, cancer	G/G 65.5	A/G 30.0	A/A 4.5	1.000

rs1800795	*IL6*	Interleukin 6	Inflammation	Obesity, CVD, diabetes, cancer	**G/G** 46.8	**C/G** 39.9	**C/C** 13.3	1.000
rs12535708	*LEP*	Leptin	Signalling, metabolism	Obesity	**C/C** 51.5	**A/C** 40.0	**A/A** 8.5	1.000
rs1137101	*LEPR*	Leptin receptor	Signalling, metabolism	Obesity, cancer	**G/G** 12.9	**A/G** 51.3	**A/A** 35.8	1.000
rs1800588	*LIPC*	Hepatic lipase C	Metabolism of lipids	CVD, diabetes	**T/T** 4.6	**C/T** 35.1	**C/C** 60.3	1.000
rs11693809	*LPIN1*	Mg(2+)-dependent phosphatidic acid phosphohydrolase	Metabolism	Diabetes, MetS	**T/T** 18.2	**C/T** 46.3	**C/C** 35.5	1.000
rs2716610	*LPIN1*	Mg(2+)-dependent PA phosphohydrolase	Metabolism	Diabetes, MetS	**T/T** 3.0	**C/T** 35.2	**C/C** 61.8	1.000
rs328	*LPL*	Lipoprotein lipase	Metabolism	CVD	**G/G** 2.7	**C/G** 24.5	**C/C** 72.8	1.000
rs17782313	*MC4R*	Melanocortin 4 receptor	Signalling, metabolism	Obesity, diabetes, cancer	**T/T** 66.2	**C/T** 30.8	**C/C** 3.0	1.000
rs1801133	*MTHFR*	Methylenetetrahydrofolate reductase	Metabolism	CVD, cancer	**G/G** 40.0	**A/G** 41.5	**A/A** 18.5	1.000
rs16139	*NPY*	Neuropeptide Y	Metabolism	Obesity	**T/T** 95.7	**C/T** 4.3	**C/C** 0.0	1.000
rs3736235	*ORL1*	Opioid receptor-like receptor	Metabolism	CVD	**T/T** 23.0	**C/T** 52.8	**C/C** 24.2	1.000
rs894160	*PLIN1*	Perilipin 1	Metabolism	Obesity	**C/C** 55.1	**C/T** 36.6	**T/T** 8.3	1.000
rs6713532	*POMC*	Proopiomelanocortin	Metabolism	Obesity	**T/T** 55.8	**C/T** 33.1	**C/C** 11.1	0.731
rs662	*PON1*	Paraoxonase 1	Lipoproteins oxidation	CVD	**C/C** 8.0	**C/T** 42.7	**T/T** 49.3	1.000
rs6008259	*PPARα*	Peroxisome proliferator-activated receptor-α	Signalling, metabolism	Cancer	**A/A** 4.9	**A/G** 27.9	**G/G** 67.2	1.000
rs1801282	*PPARγ*	Peroxisome proliferator-activated receptor-γ	Signalling, metabolism	Diabetes, cancer	**G/G** 0.5	**C/G** 17.1	**C/C** 82.4	1.000
rs2066826	*PTGS2*	Prostaglandin-endoperoxide synthase 2	Inflammation	Diabetes, cancer	**C/C** 73.7	**C/T** 25.1	**T/T** 1.1	1.000
rs3758538	*RBP4*	Retinol binding protein 4	Inflammation	Obesity	**T/T** 74.3	**G/T** 20.7	**G/G** 5.0	0.410
rs6131	*SELP*	Selectin P	Cell adhesion	CVD	**C/C** 68.3	**C/T** 28.3	**T/T** 3.4	1.000
rs11868035	*SREBF1*	Sterol regulatory element binding transcription factor 1	Metabolism	Diabetes	**G/G** 49.5	**A/G** 36.8	**A/A** 13.7	0.027
rs7903146	*TCF7L2*	Transcription factor 7-like 2	Signalling, metabolism	MetS, cancer, diabetes	**C/C** 46.7	**C/T** 42.9	**T/T** 10.4	1.000
rs1800629	*TNFα*	Tumour necrosis factor-α	Inflammation	Obesity	**G/G** 77.6	**A/G** 19.3	**A/A** 3.1	0.692
rs659366	*UCP2*	Uncoupling protein 2	Metabolism	Obesity	**T/T** 16.3	**C/T** 43.2	**C/C** 40.5	1.000

Supplementary Table 1. Description of the genotyped single-nucleotide polymorphisms (SNPs). [a]Green, favourable; Red, unfavourable; Orange, neutral; [b]The exact test of the Hardy–Weinberg equilibrium (HWE) for the normoweight individuals was calculated with the software R (HeiderWeinberg package and function 'hwexact') and Bonferroni corrected for multiple tests [1] (The SNPs rs5082, rs1801253 and rs11868035 did not satisfy the HWE, which was due to the presence of siblings. This did not affect our results because we are not estimating odds ratios of particular SNPs, but of global genetic variables generated from the MCA of all SNPs); CVD, cardiovascular disease; MetS, metabolic syndrome; TLR, Toll-like receptor; redundant predictors: rs9928094-*FTO* and rs9935401-*FTO*. The statistical model also removed SNPs with either favourable or unfavourable genotype frequencies below 5% (rs4994-*ADRB3*, rs7913948-*ALOX5*, rs7412-*APOE*, rs328-*LPL*, rs16139-*NPY*, rs6008259-*PPARα*, rs2066826-*PTGS2*).

References

[1] Graffelman, J. Exploring Diallelic Genetic Markers: The HardyWeinberg Package. *J Stat Soft.* **64**, 1-23 (2015).

Age	Mean ± SD	Median	Range
5 (*n* = 30)	7.1±2.6	7.5	(1–11)
6 (*n* = 19)	8.5±2.1*	8.5	(4–13)
7 (*n* = 25)	7.2±2.3	7.0	(2–11)
8 (*n* = 32)	6.5±1.8	6.0	(3–10)
9 (*n* = 24)	7.4±2.1	8.0	(3–13)
10 (*n* = 30)	7.4±2.2	8.0	(2–11)
11 (*n* = 37)	6.6±1.7	7.0	(1–10)
12 (*n* = 41)	6.8±2.4	6.0	(2–12)
13 (*n* = 31)	6.3±1.7[#]	6.0	(3–9)
14 (*n* = 30)	6.4±1.8	6.5	(2–9)
15 (*n* = 38)	6.3±2.2[#]	6.0	(3–11)
16 (*n* = 44)	7.0±2.2	7.0	(1–11)
17 (*n* = 34)	6.9±2.0	7.0	(3–11)
Total (*n* = 415)	6.9±2.1	7.0	(1–13)

Supplementary Table 2. KIDMED score in the population study (*n* = 415). *Significantly different ($p < 0.05$) from # (ANOVA and Bonferroni posthoc).

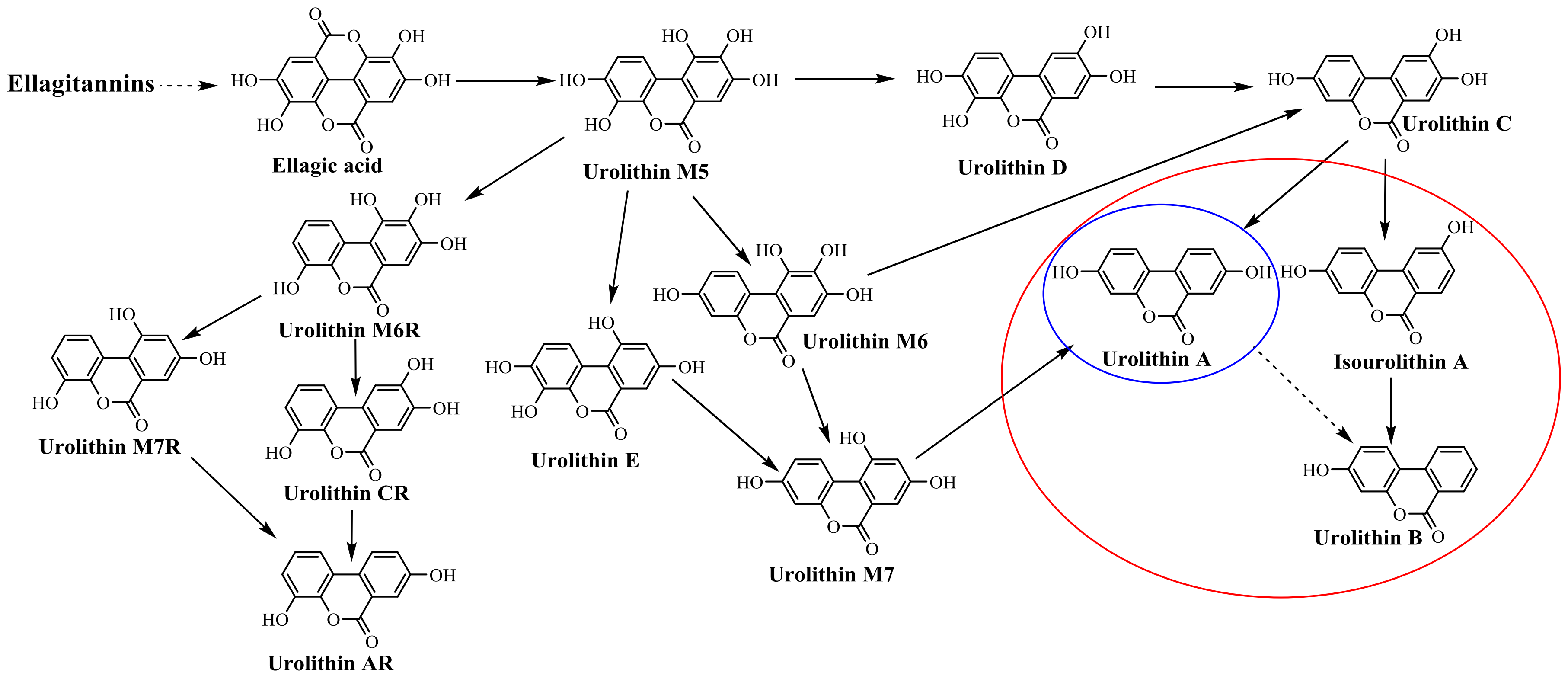

Supplementary Figure 1.* Gut microbiota-associated urolithin metabotypes resulting from the catabolism of the polyphenol ellagic acid. The circles enclose the final urolithins for each metabotype: red, urolithin metabotype B (UM-B), and blue, urolithin metabotype A (UM-A). Those individuals that cannot produce urolithins belong to the so-called 'metabotype 0' (UM-0). *Adapted from: Cortés-Martín, A., Selma, M.V., Tomás-Barberán, F.A., González-Sarrías, A., Espín, J.C. Where to Look into the Puzzle of Polyphenols and Health? The Postbiotics and the Gut Microbiota Associated with Human Metabotypes. *Mol. Nutr. Food Res*. e1900952 (2020). doi: 10.1002/mnfr.201900952.

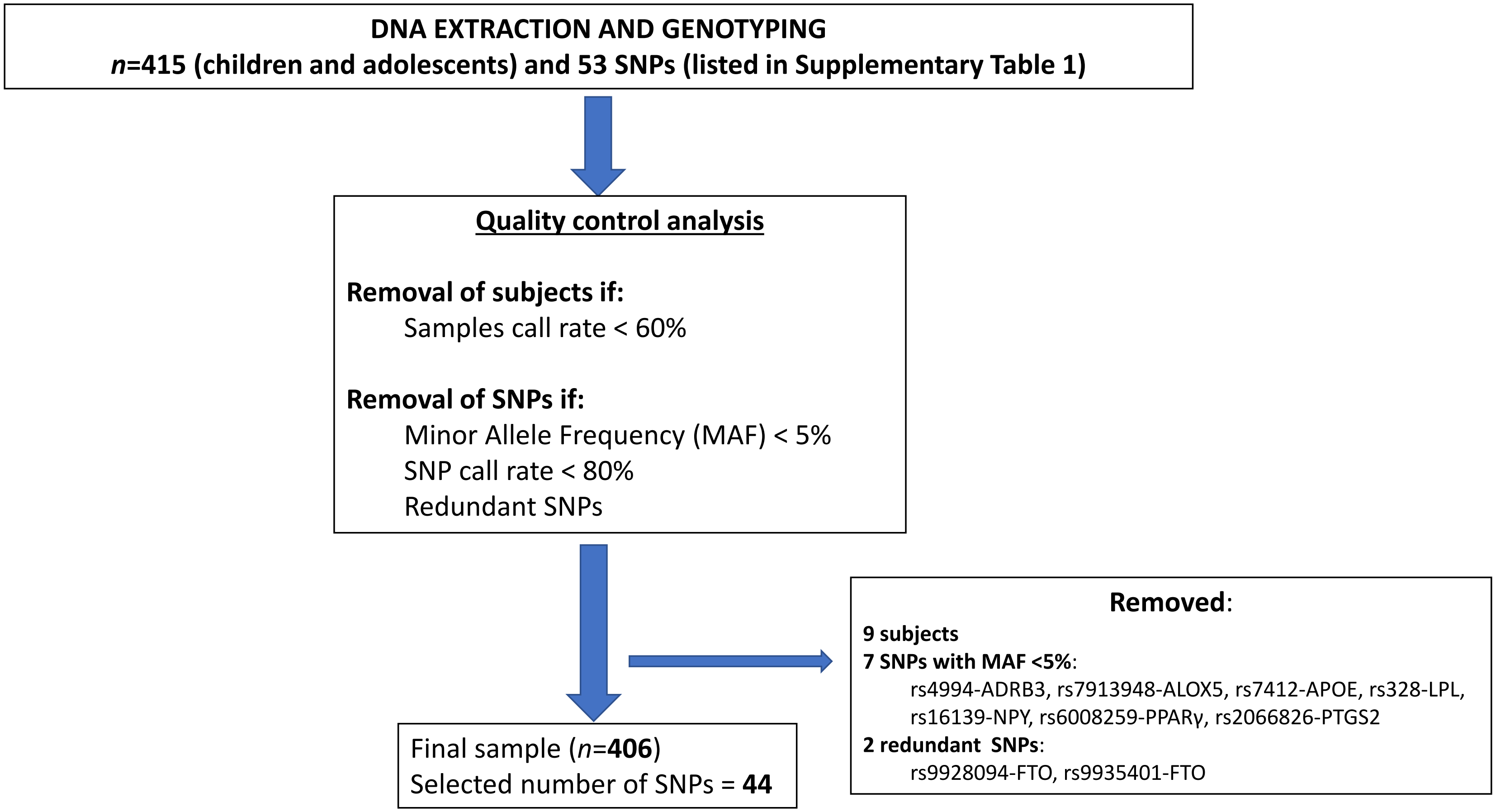

Supplementary Figure 2. Workflow of SNPs analysis in the present study.

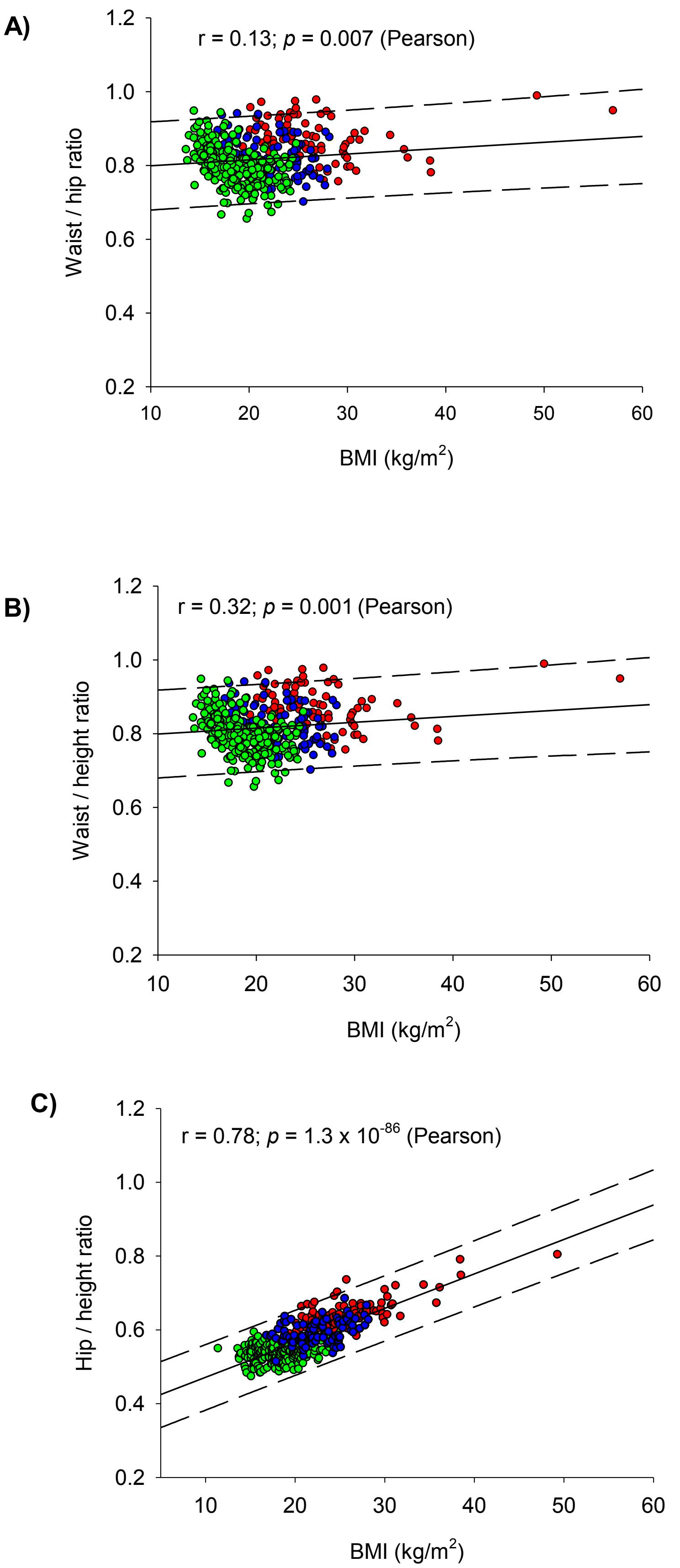

Supplementary Figure 3. Association of BMI values in children and adolescents with their (**A**) waist-to-hip ratio, (**B**) waist-to-height ratio and (**C**) hip-to-height ratio. (●) Normoweight, (●) Overweight, (●) Obesity.
